# Incidence and Radiological Spectrum of Interstitial Lung Disease in Patients With Lung Cancer Treated With Tyrosine Kinase Inhibitors: A Single-Center Observational Study

**DOI:** 10.7759/cureus.105260

**Published:** 2026-03-15

**Authors:** Supantha De, Prasanta R Mohapatra, Mahismita Patro, Sudipta Mohakud, Pritinanda Mishra, Sourin Bhuniya, Manoj Panigrahi, Shakti Bal, Mukund Sable, Aneri Parekh, Siddarth Singh, Nilava Banerjee

**Affiliations:** 1 Pulmonary Medicine and Critical Care, All India Institute of Medical Sciences, Bhubaneswar, Bhubaneswar, IND; 2 Radiology, All India Institute of Medical Sciences, Bhubaneswar, Bhubaneswar, IND; 3 Pathology and Laboratory Medicine, All India Institute of Medical Sciences, Bhubaneswar, Bhubaneswar, IND

**Keywords:** interstitial lung disease, non-small cell lung cancer, progression-free survival, real-world study, tyrosine kinase inhibitors

## Abstract

Background

Tyrosine kinase inhibitors (TKIs) have improved outcomes in driver mutation-positive non-small cell lung cancer (NSCLC), yet drug-induced interstitial lung disease (ILD) remains an uncommon but clinically relevant toxicity.

Objectives

To determine the incidence and radiological spectrum of ILD among lung cancer patients receiving TKIs, describe accompanying adverse effects, identify clinical and treatment-related risk factors, and estimate progression-free survival (PFS).

Methods

This single-center observational study included consecutive mutation-positive lung cancer patients treated with TKIs between January 2024 and September 2025 at a tertiary institute in Eastern India. Baseline demographic, clinical, radiological, and molecular data were recorded. Patients were followed for 12 months with structured clinical assessment, laboratory monitoring, and imaging. ILD was classified as TKI-attributable or all-cause.

Results

Among 106 patients, most were aged 50 years or older (80.2%), male participants (54.7%), non-smokers (85.8%), and had advanced-stage disease (93.4%). Epidermal Growth Factor Receptor (EGFR) exon19 (50.9%) and exon21 (16.0%) mutations dominated in the group. Erlotinib (48.1%) and osimertinib (22.6%) were most frequently used. TKI-induced ILD occurred in 3.7% of patients, while all-cause ILD occurred in 16.0%. Radiologically, 95.3% of the study group showed no ILD pattern. Disease control was achieved in 68.8% of cases. Mean PFS was 10.4 ± 2.91 months, with a 12-month survival of 61.2%. Mild toxicities predominated; 84.0% had no Common Terminology Criteria for Adverse Events (CTCAE)-graded toxicity at one year. Smoking status correlated with treatment response (p=0.045), and Eastern Cooperative Oncology Group (ECOG) performance predicted PFS (p=0.048). No baseline factor independently predicted ILD.

Conclusions

TKIs demonstrated favorable disease control with low attributable ILD. Continued monitoring and structured risk assessment remain essential.

## Introduction

Lung cancer remains the most frequently diagnosed form of malignancy worldwide and also the leading cause of death associated with cancer. According to the Global Cancer Observatory (GLOBOCAN) estimate for the year 2020, lung cancer comprises approximately 2.2 million new cases and 1.8 million deaths worldwide, which is equivalent to 11.4% of new cases and 18% of all cancer deaths [1]. Non-small cell lung cancer (NSCLC) contributes to approximately 85% of all lung cancers [2]. In India, it is estimated that lung cancer contributes to approximately 5.9 per 100,000 new cases of cancer and 8.1 per 100,000 deaths due to cancer each year. Patient access to treatment is mainly observed after diagnosis, when the disease is already at an advanced stage [3].

Lung cancer has been associated with clinical, psychological, and social burdens. These include symptoms of chronic cough, shortness of breath, and weakness, apart from frequent hospitalizations and side effects of medications [4]. Distress, anxiety, and depressive disorders have been demonstrated to affect one-third to half of all patients and exert a negative influence over health-related quality of life [5]. Stigma, fear of death, toxicity of treatment costs, along with uncertainties of therapy response, have been associated with heavy psychological burdens [6,7].

Conventional treatment includes platinum doublet chemotherapy and thoracic radiation according to stage of the disease. These represent a historic standard of care for patients with advanced NSCLC and show an improvement in survival duration but also trigger systemic toxicity like myelosuppression, peripheral neuropathy, mucositis, and visceral toxicity [8]. The emergence of immunotherapies and antiangiogenic treatments has significantly transformed current therapeutic approaches. Nonetheless, it is important to recognize that these therapies may be associated with adverse effects, including immune-related pneumonitis [9].

However, there has been a paradigm shift due to the identification of actionable mutations in driver genes, which has paved the way for oral tyrosine kinase inhibitors (TKIs) targeting Epidermal Growth Factor Receptor (EGFR) mutations and Anaplastic Lymphoma Kinase (ALK)/ROS oncogene 1 (ROS1) rearrangements. The superiority of targeted therapies over chemotherapy in response rate and progression-free survival (PFS) has been demonstrated in multiple studies of patients with mutated NSCLC [10]. The efficacy of the first- and second-generation TKIs in achieving median PFS of nine to 13 months in patients was shown to be inferior to that of third-generation TKIs, including osimertinib, which have proven to be more effective in terms of central nervous system permeability and survival [11]. TKIs are generally better tolerated and align with the growing emphasis on treatments that preserve quality of life while maintaining efficacy.

However, they are not without adverse effects. Common toxicities include acneiform rash, diarrhea, paronychia, and hepatotoxicity [12]. Drug-induced interstitial lung disease (DI-ILD) or pneumonitis represents one of the most serious and potentially fatal complications. EGFR TKI-associated ILD occurs in 0-5.2% of treated patients, with severe Common Terminology Criteria for Adverse Events (CTCAE) grade ≥three ILD reported in 0.6-2.2% of patients. A higher incidence is documented among East Asian populations [13]. Similar ILD patterns have been reported with ALK inhibitors and specific cytotoxic agents [14].

Given the increasing use of TKIs in India, there is a pressing need for robust real-world data to characterize the incidence, radiological patterns, and clinical determinants of TKI-associated ILD in diverse populations. This study addresses that gap by evaluating ILD in a real-world cohort of mutation-positive lung cancer patients receiving first-, second- and third-generation EGFR TKIs or ALK/ROS1 inhibitors at a tertiary care center. We hypothesized that, within this Indian population, targeted therapies would provide meaningful disease control with a relatively low incidence of TKI-attributable ILD, and that baseline clinical characteristics and performance status would influence ILD risk and other toxicities.

Accordingly, we conducted a single-center observational study to assess the incidence, clinical-radiological spectrum, and predictors of ILD among patients with lung cancer treated with TKIs, ensuring real-world representation without altering routine clinical practice.

## Materials and methods

Study design

The study was undertaken in the Department of Pulmonary Medicine and Critical Care at the All India Institute of Medical Sciences (AIIMS), Bhubaneswar, Odisha, a tertiary referral center situated in Eastern India. Both outpatients and inpatients receiving TKI treatment contributed to patient recruitment and follow-up. The study was conducted from January 2024 to September 2025, with a minimum follow-up duration of 12 months to evaluate ILD events, other adverse effects, and PFS.

Study population

Participants who fulfilled the criteria were all consecutive mutation-positive NSCLC patients undergoing TKI treatment at this hospital during the study. To participate, patients who were mutation-positive and had confirmatory testing for EGFR, ALK, or ROS1 mutations were invited. Patients who were lost to follow-up or who refused to sign informed consent were omitted.

Sample size determination

Sample size was calculated using the conventional method for proportionate studies. Given a prevalence of 6.5% for TKI-induced ILD and a confidence level of 95%, the minimum number of observed study participants required was 94. Because of anticipated attrition rates of 10% to 15% in the study, the adjusted range was 105 to 118, and this was taken into consideration along with other factors such as the accessibility and affordability of TKIs, resulting in the recruitment of 106 study participants. The incidence of lung cancer presented a substantial case load at our institution. However, the limited availability of TKIs due to their relatively high out-of-pocket costs resulted in fewer patients being eligible for treatment. Additionally, the study period was constrained to maintain the study's statistical power within a feasible range.

Ethical considerations

The study was designed to adhere to the Indian Council of Medical Research (ICMR) and Good Clinical Practice (GCP) guidelines. Consent forms in English and the local language were obtained, and confidentiality was maintained throughout the study. The Institutional Ethics Committee, All India Institute of Medical Sciences, Bhubaneswar issued approval for the study (approval no: IEC/AIIMS BBSR/PG THESIS/2023-24/147).

Data collection procedures

The baseline evaluation tools included demographic profiling, smoking history, comorbidities, presenting symptoms, physical examination, and investigations such as blood count, liver and renal function tests, ECG, and radiological studies, including chest X-ray and baseline high-resolution computed tomography (HRCT) were done. Factors in lung cancer, including histological type, mutation status, Tumor, Nodes, and Metastasis (TNM) classification, Eastern Cooperative Oncology Group (ECOG) status, and prior therapies, were evaluated.

Patients were followed up one month after initiation of the TKIs, and thereafter at three-month intervals for a year. Only patients having baseline HRCT thorax were included in the study group. In the evaluation, respiratory symptoms (dyspnea, cough, and fever), physical examination (including measurement of oxygen saturation), chest X-ray, and evaluation of skin, gastrointestinal, hepatic, and cardiovascular toxicities were performed. Yet, if there was a clinical suspicion of ILD, the review would include further investigations of the HRCT scan and its related microbiologic analysis, which comprised a sputum test, Cartridge Based Nucleic Acid Amplification Test (CBNAAT), bronchoscopy, and bronchoalveolar lavage.

Management of adverse events

The management of ILD, perceived as secondary to TKI, was performed in accordance with facility protocols. The implicated TKI was withdrawn, and systemic steroids were introduced in cases of moderate to severe ILD. The management of other adverse drug reactions was conducted in accordance with facility medical protocols and did not in any way influence the study procedures.

Study variables

Independent variables included age, sex, smoking status, baseline lung status, performance status, mutation type, and TKI administered. Outcome variables included ILD incidence and radiological patterns, other adverse effects, and PFS. Primary and secondary outcomes were predefined as: incidence and radiological spectrum of ILD; incidence and type of other toxicities; identification of risk factors for ILD; and estimation of PFS.

Statistical analysis

Data were recorded in Microsoft Excel (Microsoft Corp., Redmond, WA, USA) and analyzed using IBM SPSS Statistics for Windows, Version 30 (Released 2024; IBM Corp., Armonk, New York, United States). Descriptive statistics summarized demographic and clinical parameters. Categorical variables were compared using the chi-square test or Fisher’s exact test, as appropriate. Logistic regression was employed to examine associations between baseline characteristics and ILD development. Cox proportional hazards models were used to evaluate predictors of PFS. Kaplan-Meier survival curves were generated to estimate PFS. A p-value <0.05 was considered statistically significant.

## Results

Baseline characteristics

A total of 106 patients were analyzed. Most were ≥50 years old (85/106, 80.2%), and 58 (54.7%) were male patients. A majority of the patients were living in rural areas (82/106, 77.4%), and most of them were non-smokers (91/106, 85.8%). Comorbidities were present in 39 patients (36.8%), most frequently hypertension (23/106, 21.7%) and diabetes mellitus (16/106, 15.1%). ECOG zero to one was observed in 85 patients (80.2%). Adenocarcinoma comprised 104 of 106 cases (98.1%), advanced stage disease (IVA-IVB) was present in 99 patients (93.4%), and baseline lung function was preserved in most patients, with normal lung function test (LFT) in all (106/106, 100%) and normal kidney function test (KFT) in 104 (98.1%) patients. Brain metastases were noted in 20 patients (18.9%). Baseline characteristics of the study group are shown in Table 1.

**Table 1:** Baseline demographic and clinical characteristics of the study participants (n=106) ECOG: Eastern Cooperative Oncology Group.

Variable	Category	n (%)
Age	<50 years	21 (19.8)
	≥50 years	85 (80.2)
Sex	Male	58 (54.7)
	Female	48 (45.3)
Residence	Rural	82 (77.4)
	Urban	24 (22.6)
Smoking status	Smoker	15 (14.2)
	Non-smoker	91 (85.8)
Comorbidity present	Yes	39 (36.8)
	No	67 (63.2)
ECOG	0–1	85 (80.2)
	2	21 (19.8)
Brain metastasis	Present	20 (18.9)
	Absent	86 (81.1)
Histology	Adenocarcinoma	104 (98.1)

Molecular profile and TKI distribution

EGFR exon 19 deletion was the most frequent mutation (54/106, 50.9%), followed by EGFR EXON 21: L858R (17/106, 16.0%). ALK rearrangement was present in 23 patients (21.7%) and ROS1 in six (5.7%). Erlotinib was the most commonly prescribed TKI (51/106, 48.1%), followed by osimertinib (24/106, 22.6%) and ceritinib (18/106, 16.9%). EGFR mutations were the dominant molecular subtype, and EGFR-TKIs comprised the bulk of prescriptions. A substantial proportion of ALK-positive patients received next-generation ALK inhibitors, reflecting contemporary practice (Table 2).

**Table 2:** Mutation profile and TKI distribution (n=106) TKI: Tyrosine kinase inhibitors; EGFR: Epidermal Growth Factor Receptor; ALK: Anaplastic Lymphoma Kinase; ROS1: ROS oncogene 1.

Variable	n (%)
EGFR exon 19 deletion	54 (50.9)
EGFR EXON 21: L858R	17 (16.0)
EGFR T790M	6 (5.7)
ALK rearrangement	23 (21.7)
ROS1 rearrangement	6 (5.7)
TKIs used	
Erlotinib	51 (48.1)
Osimertinib	24 (22.6)
Ceritinib	18 (16.9)
Crizotinib	9 (8.5)
Others	4 (3.8)

Follow-up organ dysfunction and ILD outcomes

New-onset liver and kidney dysfunction increased progressively across follow-up. Abnormal LFT increased from 3/106 (2.8%) at month one to 23/106 (21.7%) at month 12. Abnormal KFT rose from 2/106 (1.9%) at month one to 25/106 (23.6%) at month 12. ILD-like symptoms including new-onset dry cough, shortness of breath, etc. were reported in 17 patients (16.0%) by 12 months, but radiologically confirmed ILD was uncommon (2/106, 1.9%). Biochemical toxicities accumulated gradually, suggesting chronic rather than early-onset TKI toxicity. Although ILD-like symptoms occurred in a minority, true radiological ILD remained rare, aligning with global incidence rates of TKI-associated ILD (Table 3).

**Table 3:** Follow-up abnormalities and interstitial lung disease (ILD) incidence (n=106) LFT: Liver function test; KFT: Kidney function test.

Time	Abnormal LFT n (%)	Abnormal KFT n (%)	ILD symptoms n (%)	Radiological ILD n (%)
1 month	3 (2.8)	2 (1.9)	3 (2.8)	1 (0.9)
3 months	9 (8.5)	7 (6.6)	9 (8.5)	1 (0.9)
6 months	17 (16.0)	12 (11.3)	11 (10.4)	2 (1.9)
9 months	20 (18.9)	17 (16.0)	16 (15.1)	2 (1.9)
12 months	23 (21.7)	25 (23.6)	17 (16.0)	2 (1.9)

Adverse events

Rash and diarrhea were the most prevalent early toxicities. At month one, rash was observed in 32/106 (30.2%) of the study group. It was the most common adverse event and persisted with skin manifestations throughout the study period. But it was self-limiting or tolerable or was treated with topical steroids. Systemic therapies were seldom required. The second most common adverse event encountered was diarrhea in 20/106 (18.8%). Toxicity burden reduced considerably over time; by month 12, 71/106 (67.0%) reported no adverse events. Early cutaneous and gastrointestinal toxicities were common but predominantly mild and self-limiting. Late toxicities were also frequent, and the majority remained toxicity-free at nine months, although at 12 months some patients manifested late-onset toxicity that were mostly manageable, demonstrating good long-term tolerability (Table 4).

**Table 4:** Treatment-emergent adverse events over time (n=106)

Event	1 month; n (%)	3 months; n (%)	6 months; n (%)	9 months; n (%)	12 months; n (%)
Rash	32 (30.2)	37 (34.9)	21 (19.8)	15 (14.2)	5 (4.7)
Diarrhea	20 (18.8)	18 (16.9)	12 (11.3)	6 (5.7)	2 (1.9)
Fatigue	9 (8.5)	13 (12.3)	8 (7.5)	7 (6.6)	4 (3.8)
Dyslipidemias	8 (7.5)	11 (10.4)	7 (6.6)	5 (4.7)	2 (1.9)
Transaminitis	3 (2.8)	9 (8.5)	8 (7.5)	5 (4.7)	3 (2.8)
None	30 (28.3)	23 (21.7)	60 (56.6)	88 (83.0)	71 (67.0)

Tumor response and survival

Partial response was achieved in 26/106 (24.5%), stable disease in 47/106 (44.3%), and progressive disease in 31/106 (29.2%), yielding a disease control rate of 68.8%. Complete response occurred in 2/106 (1.9%). Mean PFS was 10.4 ± 2.91 months. Kaplan-Meier survival probability was 0.612 at 12 months. Approximately two-thirds of patients achieved disease control on TKI therapy, and PFS was consistent with real-world and clinical-trial data for EGFR-mutated and ALK-rearranged NSCLC (Table 5).

**Table 5:** Tumour response (RECIST) (n=106) RECIST: Response Evaluation Criteria in Solid Tumors.

Response	n (%)
Complete response	2 (1.9)
Partial response	26 (24.5)
Stable disease	47 (44.3)
Progressive disease	31 (29.2)

Predictors of ILD and treatment outcomes

TKI-induced ILD occurred in 4/106 (3.7%). ILD occurrence varied significantly by TKI type (p=0.011), with a higher incidence among erlotinib (two patients developed ILD), osimertinib (one patient developed ILD), and crizotinib (one patient developed ILD) users. Smoking status was significantly associated with treatment response (p=0.045), and ECOG performance status correlated substantially with PFS (p=0.048). Drug-specific ILD risk was evident, consistent with the differential pneumonitis profiles of various TKIs. Non-smokers demonstrated better tumor response, and baseline performance status remained a strong determinant of PFS (Table 6).

**Table 6:** Significant associations TKI: Tyrosine kinase inhibitor; ILD: Interstitial lung disease; ECOG: Eastern Cooperative Oncology Group; PFS: Progression-free survival.

Variable	Association	p-value
TKI type vs ILD incidence	Significant	0.011
Smoking vs poor response	Significant	0.045
ECOG vs PFS	Significant	0.048

## Discussion

The present study evaluated the incidence, clinical-radiological profile, and risk determinants of ILD among patients with mutation-positive lung cancer receiving TKIs. Our findings demonstrated a low rate of confirmed TKI-induced ILD and a high overall disease-control rate in a real-world Indian cohort, reflecting the favorable benefit-toxicity balance seen with modern targeted therapy. Given the rising burden of EGFR-mutated and ALK-rearranged NSCLC in Asian populations, real-world evidence from diverse settings is essential to understand patterns of toxicity, response, and survival beyond clinical trials. Background literature was the literature searched for in databases.

The main objective was to assess the incidence rate of TKI-induced ILD. Our results closely match those reported in Asian post-marketing surveillance studies and meta-analyses. Japanese sentinel surveillance studies have reported ILD rates ranging from 1-5% with the first-generation EGFR TKIs gefitinib and erlotinib [13]. Other real-world studies and meta-analyses reported that the overall ILD risk secondary to TKI therapy was 1-4%, with a greater risk for elderly patients, smokers, and patients presenting with initial lung injury [15,16]. The findings in our study, that mild radiographic patterns (three patients had reticular patterns and one patient had an unusual interstitial pneumonia-like pattern) and late-onset ILD were dominant, are in keeping with those reported by Camus et al. [17], who found that ILD secondary to TKI therapy for NSCLC was more frequently characterized by features of pure diffuse alveolar damage (DAD) or non-specific interstitial pneumonia (NISP) rather than more fulminant acute respiratory distress syndrome (ARDS) patterns. The slightly elevated ILD signal seen in patients treated with erlotinib, osimertinib, and crizotinib can be biologically explained by noting that these medications are associated with a higher risk of pneumonitis in various studies [18,19]. Biological theories also indicate that the presence or absence of molecular selectivity, or of various off-target signaling components, is essential for achieving different outcomes. The relatively low signal observed in our series may also reflect early detection and enhanced awareness, as advocated in the ILD safety communications in the literature.

Our secondary objectives included treatment response, PFS, and patterns of adverse events. Our disease control (DC) rate and median PFS (M-PFS) value were comparable to those reported in extensive studies of NSCLC patients treated with TKIs, including the Iressa Pan-ASia Study (IPASS) study [20] and the AZD9291 Under Resistance Approach 3 (AURA3) study investigating osimertinib [19], which demonstrated a beneficial response with improvements in PFS. Other real-world studies among Asian populations also yielded comparable findings regarding the role of treatment effectiveness, which paralleled the associations demonstrated in our study [21], especially among non-smokers with good ECOG performance scores. The adverse events described in our study are comparable to those in other TKI studies, including those observed in the First-line Treatment of EGFR-Mutated Advanced Non-Small-Cell Lung Cancer (FLAURA) study [11]. The progressive accumulation of biochemical aberrancies with prolonged treatment has also been observed in extensive safety studies, including third-generation TKIs for EGFR and a first-generation TKI.

Briefly, these findings confirm the benefits of TKIs in Indian patients with mutated NSCLC and underscore the importance of ILD monitoring. The early diagnosis of ILD is crucial for the best outcomes in a real-world Indian setting with a high comorbidity index and several environmental factors, and where diagnosis can often be delayed. The remarkably reduced incidence of severe ILD in this study also indicates the safety of TKIs in conjunction with careful monitoring. However, several limitations in the design and conduct of this study exist. The major limitation is that this is a hospital-based observational study. A relatively small sample size limits the dataset. The study does not provide histological confirmation, although the recent guidelines suggest multidisciplinary discussion for the diagnosis of ILD [22-24]. This study is still significant within the development of real-world findings.

## Conclusions

In this study, TKIs demonstrated a favorable efficacy profile in patients with mutation-positive NSCLC, with a low incidence of TKI-induced ILD. The most observed side effects were mild, with few significant adverse events leading to therapy discontinuation. These findings support the established effectiveness and manageable safety profile of TKIs within this molecularly selected population, highlighting the importance of continued monitoring for ILD.

The lack of reliable clinical predictors for ILD onset emphasizes the unpredictable nature of this uncommon but serious complication, underscoring the necessity for vigilant surveillance across all patients rather than risk-based stratification. Overall, the benefits of TKI therapy remain substantial, and the low rate of ILD supports their continued use as a key treatment modality in mutation-positive NSCLC.
